# A Comparison of Pneumococcal Nasopharyngeal Carriage in Very Young Fijian Infants Born by Vaginal or Cesarean Delivery

**DOI:** 10.1001/jamanetworkopen.2019.13650

**Published:** 2019-10-18

**Authors:** Eleanor Frances Georgina Neal, Cattram Nguyen, Felista Tupou Ratu, Silivia Matanitobua, Eileen Margaret Dunne, Rita Reyburn, Mike Kama, Rachel Devi, Kylie M. Jenkins, Lisi Tikoduadua, Joseph Kado, Eric Rafai, Catherine Satzke, Edward Kim Mulholland, Fiona Mary Russell

**Affiliations:** 1 Infection and Immunity, Murdoch Children’s Research Institute, Royal Children’s Hospital, Parkville, Victoria, Australia; 2 Department of Paediatrics, The University of Melbourne, Parkville, Victoria, Australia; 3 Centre for International Child Health, Department of Paediatrics, The University of Melbourne, Parkville, Victoria, Australia; 4 Ministry of Health and Medical Services, Suva, Fiji; 5 Fiji Health Sector Support Program, Suva, Fiji; 6 College of Medicine Nursing and Health Sciences, Fiji National University, Suva, Fiji; 7 Telethon Kids Institute, University of Western Australia, Nedlands, Western Australia, Australia; 8 Department of Microbiology and Immunology, Peter Doherty Institute for Infection and Immunity, The University of Melbourne, Parkville, Victoria, Australia; 9 Department of Infectious Disease Epidemiology, London School of Hygiene and Tropical Medicine, London, United Kingdom

## Abstract

**Importance:**

Pneumococcal nasopharyngeal carriage is a prerequisite for pneumococcal disease. The main transmission route is from toddlers, who commonly carry pneumococci. However, neonatal pneumococcal disease case reports suggest that vertical pneumococcal transmission may also occur.

**Objective:**

To describe and compare pneumococcal nasopharyngeal carriage and density by infant mode of delivery in young Fijian infants.

**Design, Setting, and Participants:**

Annual cross-sectional surveys were performed in Suva, Fiji, before the introduction of 10-valent pneumococcal conjugate vaccine (PCV10), from September 14 to December 20, 2012, and after PCV10 was introduced, from July 11 to November 19, 2013; July 15 to December 9, 2014; and August 13 to November 19, 2015. Caregivers of 2006 infants aged 5 to 8 weeks participated in the surveys. Statistical analysis was performed from May 24, 2018, to August 12, 2019.

**Exposures:**

Caregivers provided data on infant mode of delivery and demographics via interviewer-led survey.

**Main Outcomes and Measures:**

Pneumococci in swab samples were detected and quantified by *lyt*A quantitative polymerase chain reaction with molecular serotyping by microarray. Pneumococci were categorized as PCV10 or non-PCV10 serotypes.

**Results:**

Of the 2006 infants (976 girls and 1030 boys; mean [SD] age, 6.1 [0.02] weeks]), 1742 (86.8%) were born vaginally and 264 were born by cesarean delivery. Infants delivered vaginally, compared with those born by cesarean delivery, had a higher prevalence of overall pneumococcal nasopharyngeal carriage (470 of 1722 [27.3%; 95% CI, 25.2%-29.4%] vs 47 of 260 [18.1%; 95% CI, 13.6%-23.3%]; *P* = .002), PCV10 carriage (113 of 1698 [6.7%; 95% CI, 5.5%-7.9%] vs 8 of 256 [3.1%; 95% CI, 1.4%-6.1%]; *P* = .03), and non-PCV10 carriage (355 of 1698 [20.9%; 95% CI, 19.0%-22.9%] vs 38 of 256 [14.8%; 95% CI, 10.7%-19.8%]; *P* = .02), and had higher median densities of overall pneumococci (4.9 log_10_ genome equivalents [GE]/mL [interquartile range, 4.8-5.0 log_10_ GE/mL] vs 4.5 log_10_ GE/mL [interquartile range, 4.1-4.6 log_10_ GE/mL]; *P* = .01) and non-PCV10 pneumococci (4.9 log_10_ GE/mL [interquartile range, 4.7-5.0 log_10_ GE/mL] vs 4.4 log_10_ GE/mL [interquartile range, 4.0-4.7 log_10_ GE/mL]; *P* = .01). Vaginal delivery was associated with overall (adjusted odds ratio, 1.57 [95% CI, 1.10-2.23]; *P* = .01) and non-PCV10 (adjusted odds ratio, 1.49 [95% CI, 1.01-2.20]; *P* = .04]) pneumococcal nasopharyngeal carriage. Vaginal delivery was not associated with PCV10 carriage (adjusted odds ratio, 1.67 [95% CI, 0.80-3.51]; *P* = .17). After adjustment, vaginal delivery was not associated with density.

**Conclusions and Relevance:**

Pneumococcal nasopharyngeal carriage prevalence and density were higher in infants delivered vaginally compared with those delivered by cesarean birth. After adjustment, vaginal delivery was associated with pneumococcal carriage. Differences in carriage by mode of delivery may be due to differential exposure to the vaginal microbiota during delivery and the effect of intrapartum antibiotics during cesarean delivery on the infant microbiome. Our findings also raise the hypothesis that vertical transmission may contribute to pneumococcal acquisition.

## Key Points

**Question:**

Is pneumococcal nasopharyngeal carriage associated with mode of delivery in Fijian infants aged 5 to 8 weeks?

**Findings:**

In this cross-sectional study, pneumococcal nasopharyngeal carriage prevalence, density, and serotype range were higher in infants delivered vaginally vs cesarean delivery. After adjustment, vaginal delivery was positively associated with pneumococcal nasopharyngeal carriage.

**Meaning:**

These findings may be owing to differential exposure to the vaginal microbiota during delivery and the association of intrapartum antibiotics with the infant microbiome, and raises the hypothesis of vertical transmission.

## Teaser

This cross-sectional study uses surveys to describe and compare pneumococcal nasopharyngeal carriage and density by vaginal vs cesarean delivery in young Fijian infants.

## Introduction

Pneumococcal disease is a leading cause of global morbidity and mortality in children younger than 5 years, with the highest burden in low- and middle-income countries (LMICs).^1^ Of all pneumococcal meningitis cases in children younger than 5 years, up to 21% occur in infants younger than 2 months.^2^ A review of the causes of community-acquired neonatal sepsis in LMICs reported that the pneumococcus was isolated in 4.6% (95% CI, 2.1%-7.1%) of cases among infants in the first week of life.^3^ Neonatal (infants younger than 28 days) invasive pneumococcal disease (IPD) case fatality rates are as high as 19% in high-income settings,^4^ and approximately 50% in LMICs.^5^ Pneumococcal transmission during vaginal delivery (vertical transmission) may play a role in neonatal IPD, with maternal pneumococcal vaginal colonization present in 25 of 43 reported neonatal IPD cases (58.1%).^6,7,8,9,10,11,12,13,14,15,16,17,18,19,20,21^ Only 2 studies have published rates of pneumococcal vaginal carriage. In France, pneumococcal isolates were found in 0.66% of 1064 vaginal swabs, and in England, pneumococcal isolates were detected in 0.03% of 15 000 high vaginal, cervical, urethral, and genital swabs.^16,22^ To our knowledge, there are no published studies reporting the prevalence of pneumococcal vaginal carriage in pregnant women from LMICs, despite pneumococci being a common cause of neonatal sepsis.^3^

Nasopharyngeal pneumococcal carriage is associated with an increased risk of pneumococcal disease, and high pneumococcal nasopharyngeal load (density) is associated with severe pneumococcal pneumonia.^23,24^ In LMICs, the first acquisition of pneumococci occurs early in life. In Papua New Guinea, 59% of infants carry pneumococci by 30 days of age.^25^ Factors associated with pneumococcal carriage in young infants from LMICs remain unclear. Although contact with toddlers and children is a risk factor for pneumococcal carriage via horizontal transmission, maternal vaginal colonization, prolonged rupture of membranes, and premature delivery may be risk factors for vertical transmission.^26,27,28,29^ To our knowledge, no published studies describe infant pneumococcal carriage and density by infant mode of delivery or document the association of mode of delivery with carriage.

A previous study in Fiji described the association of 10-valent pneumococcal conjugate vaccine (PCV10) with pneumococcal carriage, which included infants aged 5 to 8 weeks who were not vaccinated for PCV10.^30^ This study aims to compare the prevalence and density of pneumococcal carriage by infant mode of delivery and to describe any associations between vaginal delivery and pneumococcal carriage and density in very young unvaccinated Fijian infants. We hypothesize that in this population, pneumococcal carriage and density differ by mode of delivery, and that vaginal delivery is associated with pneumococcal carriage.

## Methods

Fiji is a middle-income nation in the South Pacific Ocean. The population comprises 56.8% indigenous iTaukei individuals and 37.8% Fijian of Indian descent individuals. This study was undertaken in the Suva-Nausori corridor in the central division of Fiji, where approximately one-third of Fijian individuals live.^31^ Fiji introduced PCV10 in October 2012 as a 3 + 0 schedule, given at 6, 10, and 14 weeks of age, with no catch-up campaign. National coverage of the third dose of PCV10 was 84.9% 1 year after introduction, 84.9% 2 years after introduction, and 89.0% 3 years after introduction.^32,33,34^ This study was conducted according to protocols reviewed and approved by the Fiji National Research Ethics Review Committee and the University of Melbourne Human Research Ethics Subcommittee. Study staff discussed the study with parents and guardians (caregivers), who completed written informed consent prior to any study procedures. Participants and caregivers were offered no incentive to participate. This study followed the Strengthening the Reporting of Observational Studies in Epidemiology (STROBE) reporting guideline.

The study design has been described previously.^30^ Briefly, annual cross-sectional community-based nasopharyngeal carriage surveys were conducted before the introduction of PCV10 (September 14 to December 20, 2012) and after the introduction of PCV10 (July 11 to November 19, 2013; July 15 to December 9, 2014; and August 13 to November 19, 2015).^30^ We used purposive convenience sampling to ensure that the sample represented the iTaukei to Fijian of Indian descent ethnicity ratio (3:2), and rural to urban residential location ratio (1:1), as these were considered likely to be associated with pneumococcal carriage.^35^

### Participants

Infants aged 5 to 8 weeks were recruited while attending maternal and child health clinics for routine immunization at the 2 largest health centers in Suva. Inclusion criteria were age and the mother having lived in the area for 3 months or more at the time of survey. We excluded infants with an axillary temperature higher than 37.0°C or who had received any prior PCV10 doses. The sample size was calculated to answer the primary research question regarding the direct association of PCV10 with pneumococcal nasopharyngeal carriage.^30^ For convenience, this study used the same sample.

### Data Collection

Fieldworkers interviewed caregivers via questionnaire to obtain individual-level infant characteristics, including ethnicity (iTaukei or Fijian of Indian descent), sex, residential location, antibiotic use in the 2 weeks preceding the survey, infant mode of delivery, current breastfeeding status, exposure to household cigarette smoke, weekly family income, number of children younger than 5 years living in the household, and symptoms of an upper respiratory tract infection. Caregivers reported ethnicity according to investigator defined options. Ethnicity was assessed in the broader PCV10 evaluation, as it is known to be associated with pneumococcal carriage in Fiji.^35^

Nasopharyngeal samples were collected using flocked nylon swabs (FLOQSwabs; COPAN Diagnostics Inc), placed in skim milk tryptone glucose glycerol media, and stored according to standard methods.^36^ Samples were analyzed at the Murdoch Children’s Research Institute, Melbourne, Australia. The detection and density of pneumococci were determined by real-time quantitative polymerase chain reaction with primers and probes targeting the *lyt*A gene (GenBank 933669), and genomic DNA from a reference isolate as a standard.^37^ Molecular serotyping of pneumococci was conducted by microarray, as described previously.^38^

Overall pneumococcal carriage was defined as the detection of any pneumococcus by quantitative polymerase chain reaction. Carriage of PCV10 was defined as detection of PCV10 serotypes (1, 4, 5, 6B, 7F, 9V, 14, 18C, 19F, and 23F). Non-PCV10 carriage was defined as detection of serotypes not included in PCV10, including nonencapsulated pneumococci. Serotypes 15B and 15C were reported as *15B/C*, as these serotypes can interconvert.^39^ Serotype 11A was identified as *11F-like*, as reported previously.^40^

Detection of PCV10 and non-PCV10 serotypes from the same sample was recorded as positive for PCV10 and non-PCV10 carriage. Carriage of multiple serotypes was defined as detection of 2 or more serotypes from the same sample. Pneumococcal density was reported in genome equivalents (GE) per milliliter. Serotype-specific carriage was defined as detection of individual serotypes, including nonencapsulated pneumococci.

Double entry and validation of participant characteristic data were performed in EpiData, version 3.1 (EpiData). Pneumococcal data were entered into Microsoft Excel 2013 (Microsoft Corp). Participant characteristics and pneumococcal data were merged and cleaned in Stata, version 15.1 (StataCorp).

### Statistical Analysis

Statistical analyses were performed from May 24, 2018, to August 12, 2019, in Stata, version 15.1. Participant characteristics were summarized by counts and percentages, or means and SDs. Carriage data were reported as counts and percentages with 95% CIs. Pneumococcal densities were log_10_ transformed, and reported as median log_10_ GE per milliliter with interquartile range (IQR). Categorical variables were compared by the χ^2^ test. Means of continuous variables were compared using the *t* test and medians of continuous variables were compared using the Mann-Whitney test. Description of multiple-serotype carriage and analyses of serotype-specific carriage and pneumococcal density were restricted to pneumococcal carriers. We used the Fisher exact test to compare serotype-specific carriage prevalence by infant mode of delivery. All *P* values were from 2-sided tests and results were deemed statistically significant at *P* < .05.

Logistic and quantile regression models were built to determine the association of infant mode of delivery with overall, PCV10, and non-PCV10 pneumococcal carriage and density. Multivariable models adjusted for potential confounders (survey year, residential location, low family income, and ≥2 children younger than 5 years living in the household) selected a priori using a directed acyclic graph (eFigure and eAppendix in the Supplement). Correlation between exposures included in regression models was assessed using Pearson *r*.

Although ethnicity is associated with pneumococcal carriage in Fiji, it was not included in multivariable models, as infant mode of delivery does not differ by ethnicity in Fiji.^41^ The associations between infant mode of delivery with carriage were reported as odds ratios (ORs) and the associations between infant mode of delivery with density were reported as differences in medians, with 95% CIs and *P* values.

## Results

### Demographics

Of the 2006 infants, 1742 (86.8%) were born vaginally and 264 were born by cesarean delivery. Participant mean (SD) age was 6.1 (0.02) weeks (Table 1).^42^ The distribution by sex was approximately equal (976 girls and 1030 boys). However, more infants born vaginally were female, compared with those born by cesarean delivery (865 [49.7%] vs 111 [42.0%]; *P* = .02). Few participants used antibiotics in the 2 weeks preceding the survey (42 of 2005 [2.1%]). The percentage of infants living in a rural location was lower in those born vaginally compared with those born by cesarean delivery (851 [48.9%] vs 176 [66.7%]; *P* < .001). A lower percentage of infants delivered vaginally had low family income compared with those born by cesarean delivery (845 of 1697 [49.8%] vs 149 of 257 [58.0%]; *P* = .01). A higher percentage of infants born vaginally lived with children younger than 5 years compared with infants born by cesarean delivery (797 [45.8%] vs 90 [34.1%]; *P* < .001). The percentage of vaginal and cesarean delivered participants differed annually (Table 1).^42^

**Table 1.** Characteristics of Fijian Infants Aged 5 to 8 Weeks in 4 Annual Pneumococcal Nasopharyngeal Carriage Surveys

Characteristics	Infants, No./Total No. (%)			*P* Value^a^
	Total	Delivery		
		Vaginal	Cesarean	
Age, mean (SD), wk	6.1 (0.02)	6.1 (0.02)	6.1 (0.04)	.76
Female sex	976/2006 (48.7)	865/1742 (49.7)	111/264 (42.0)	.02
Ethnicity				
Fijian of Indian descent	796/2006 (39.7)	681/1742 (39.1)	115/264 (43.6)	.23
iTaukei	1202/2006 (59.9)	1053/1742 (60.4)	149/264 (56.4)	
Other	8/2006 (0.4)	8/1742 (0.5)	0	
Breastfeeding at time of survey	1857/2005 (92.6)	1609/1741 (92.4)	248/264 (93.9)	.38
Symptoms of URTI	311/2005 (15.5)	260/1742 (14.9)	51/264 (19.3)	.07
Antibiotic use in past 2 wk	42/2005 (2.1)	35/1741 (2.0)	7/264 (2.7)	.50
Residential location				
Rural	1027/2006 (51.2)	851/1742 (48.9)	176/264 (66.7)	<.001
Urban	979/2006 (48.8)	891/1742 (51.1)	88/264 (33.3)	
Low family income^b^	994/1954^c^ (50.9)	845/1697 (49.8)	149/257 (58.0)	.01
Exposure to household cigarette smoke	1011/2006 (50.4)	871/1742 (50.0)	140/264 (53.0)	.36
≥2 Children aged <5 y in household	887/2006 (44.2)	797/1742 (45.8)	90/264 (34.1)	<.001
Survey year				
Before introduction of PCV10	499/2006 (24.9)	456/1742 (26.2)	43/264 (16.3)	<.001
After introduction of PCV10				
1 y	510/2006 (25.4)	457/1742 (26.2)	53/264 (20.1)	
2 y	500/2006 (24.9)	405/1742 (23.3)	95/264 (36.0)	
3 y	497/2006 (24.8)	424/1742 (24.3)	73/264 (27.7)	
Pneumococcal carriage, No./total No. (%) [95% CI, %]				
Overall^d^	517/1982 (26.1) [24.2-28.1]	470/1722 (27.3) [25.2-29.4]	47/260 (18.1) [13.6-23.3]	.002
PCV10 serotype^e^	121/1954 (6.2) [5.2-7.4]	113/1698 (6.7) [5.5-7.9]	8/256 (3.1) [1.4-6.1]	.03
Non-PCV10 serotype^f^	393/1954 (20.1) [18.4-22.0]	355/1698 (20.9) [19.0-22.9]	38/256 (14.8) [10.7-19.8]	.02
Multiple-serotype carriage^g^	79/489 (16.2 [13.0-19.7])	72/446 (16.1 [12.9-19.9])	7/43 (16.3 [6.8-30.7])	.98
Pneumococcal density^g^				
Overall				
No.	516	469	47	.01
log_10_ GE/mL, median (IQR)	4.9 (4.7-5.0)	4.9 (4.8-5.0)	4.5 (4.1-4.6)	
PCV10 serotype				
No.	121	113	8	.39
log_10_ GE/mL, median (IQR)	5.0 (4.6-5.1)	5.0 (4.7-5.2)	4.5 (3.6-5.9)	
Non-PCV10 serotype				
No.	393	355	38	.01
log_10_ GE/mL, median (IQR)	4.8 (4.6-5.0)	4.9 (4.7-5.0)	4.4 (4.0-4.7)	

Abbreviations: GE, genome equivalents; IQR, interquartile range; PCV10, 10-valent pneumococcal conjugate vaccine; URTI, upper respiratory tract infection.

^a^ Determined from χ^2^ for categorical variables and from *t* test or Mann-Whitney test for continuous variables, as appropriate.

^b^ Weekly family income below the basic needs poverty line (<FJ$175 per week).^42^

^c^ Family income data was missing for 52 participants (2.6%).

^d^ Any pneumococci, including nonencapsulated pneumococci.

^e^ Pneumococci included in PCV10 (serotypes 1, 4, 5, 6B, 7F, 9V, 14, 18C, 19F, and 23F).

^f^ Pneumococcal serotypes not included in PCV10, including nonencapsulated pneumococci.

^g^ Only includes participants who were pneumococcal carriers, PCV10 serotype carriers, or non-PCV10 serotypes carriers.

### Pneumococcal Carriage Prevalence and Median Density

Twenty nasopharyngeal swab samples from infants delivered vaginally and 4 from infants born by cesarean delivery were excluded from analysis owing to insufficient volume, sample loss, or labeling errors. Twenty-eight pneumococcal-positive samples were excluded from serotyping owing to technical issues. Infants born vaginally had a higher prevalence of overall pneumococci (470 of 1722 [27.3%; 95% CI, 25.2%-29.4%] vs 47 of 260 [18.1%; 95% CI, 13.6%-23.3%]; *P* = .002), PCV10 pneumococci (113 of 1698 [6.7%; 95% CI, 5.5%-7.9%] vs 8 of 256 [3.1%; 95% CI, 1.4%-6.1%]; *P* = .03), and non-PCV10 pneumococci (355 of 1698 [20.9%; 95% CI, 19.0%-22.9%] vs 38 of 256 [14.8%; 95% CI, 10.7%-19.8%]; *P* = .02) compared with infants born by cesarean delivery (Table 1).^42^ Among pneumococcal carriers, the prevalence of multiple-serotype carriage was similar by infant mode of delivery. Vaginally delivered infants, compared with those born by cesarean delivery, had higher median density of overall pneumococci (4.9 log_10_ GE/mL [IQR, 4.8-5.0 log_10_ GE/mL] vs 4.5 log_10_ GE/mL [IQR, 4.1-4.6 log_10_ GE/mL]; *P* = .01) and non-PCV10 pneumococci (4.9 log_10_ GE/mL [IQR 4.7-5.0 log_10_ GE/mL] vs 4.4 log_10_ GE/mL [IQR 4.0-4.7 log_10_ GE/mL]; *P* = .01) (Table 1).^42^

### Serotype-Specific Carriage

The Figure displays serotype-specific carriage prevalence by infant mode of delivery. Of the serotypes carried by cesarean-born infants, 12.5% (3 of 24) were PCV10 serotype and 20.8% (5 of 24) were PCV13 serotype. For infants delivered vaginally, 17.6% (9 of 51) were PCV10 serotype and 21.6% (11 of 51) were PCV13 serotype. We detected PCV10 serotypes 6B, 19F, and 23F from infants born by either mode of delivery, while PCV10 serotypes 1, 4, 7F, 9V, 14, and 18C were detected from infants born only by vaginal delivery. Serotypes 4 and 18C represented 9.3% (11 of 118) of PCV10 serotypes found in such infants. Non-PCV10 serotypes 6A, 6C, 6D, 7C, 10B, 11A, 13, 15B/C, 16F, 17F, 19A, 21, 22F, 24B, 33B, 34, 35B, 38, and nonencapsulated lineages NT2, NT2/NT3b, and NT3b were detected in infants born by either mode of delivery. An additional 21 non-PCV10 serotypes were detected exclusively from infants born vaginally, including 3, 7B, 8, 10A, 12F, 15A, 18A, 19B, 20, 20B, 23A, 23B, 24F, 29, 31, 33A, 33F, 35A, 35F, 39, and the nonencapsulated lineage NT4b. NT2, NT2/NT3b, NT3b, and NT4 are genetic variants of nonencapsulated pneumococci identified by microarray.^43^ Of the 21 11A serotypes identified, 19 were genetic variants identified by microarray as 11F-like.^40^ Serotype-specific carriage prevalence did not differ by infant mode of delivery (eTable 1 in the Supplement).

**Figure.**
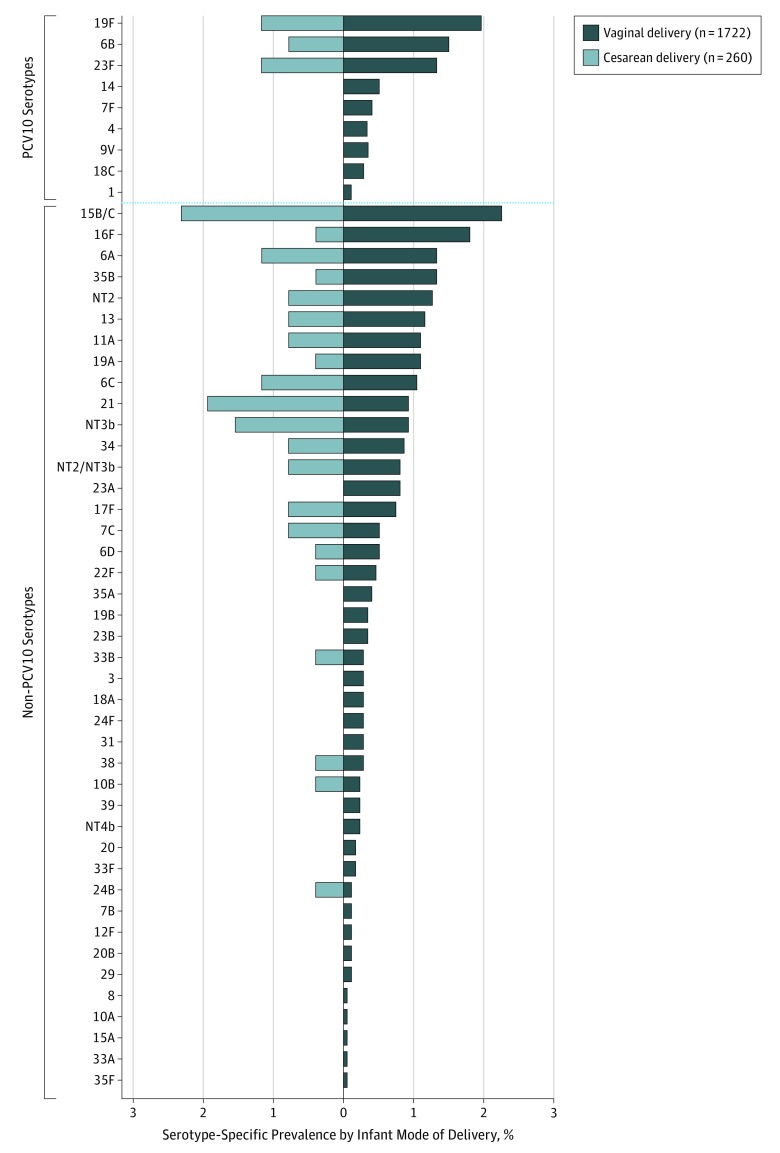
Pneumococcal Serotype-Specific Prevalence Among 2006 Fijian Infants, by Infant Mode of Delivery The total sample in the study was 2006: 1742 infants delivered vaginally and 264 born via cesarean delivery. A total of 24 nasopharyngeal swab samples were unable to be processed for pneumococcal detection: 20 from infants born vaginally and 4 from infants born by cesarean delivery. For pneumococcal results, only 1722 vaginally delivered infants and 260 born via cesarean delivery had results for carriage. PCV10 indicates 10-valent pneumococcal conjugate vaccine.

### Association Between Infant Mode of Delivery and Pneumococcal Carriage

Table 2 shows the association of infant mode of delivery with pneumococcal carriage (overall, PCV10, and non-PCV10). Vaginal delivery was positively associated with overall pneumococcal nasopharyngeal carriage (adjusted OR [aOR], 1.57 [95% CI, 1.10-2.23]; *P* = .01) and non-PCV10 carriage (aOR, 1.49 [95% CI, 1.01-2.20]; *P* = .04). Vaginal delivery was not associated with PCV10 carriage (aOR, 1.67 [95% CI, 0.80-3.51]; *P* = .17). There was no evidence of correlation between exposure variables included in regression models (eTable 2 in the Supplement).

**Table 2.** Associations Between Infant Mode of Delivery and Pneumococcal Carriage in Fijian Infants Aged 5 to 8 Weeks, in 4 Annual Pneumococcal Carriage Surveys

Characteristics	Unadjusted Model		Adjusted Model^a^	
	OR (95% CI)	*P* Value	OR (95% CI)	*P* Value
**Overall Pneumococcal Carriage (n = 1982)**^**b**^				
Infant mode of delivery				
Cesarean	1 [Reference]	.002	1 [Reference]	.01
Vaginal	1.70 (1.22-2.37)		1.57 (1.10-2.23)	
Survey year				
Before introduction of PCV10	1 [Reference]	<.001	1 [Reference]	<.001
After introduction of PCV10				
1 y	0.71 (0.54-0.95)		0.94 (0.69-1.27)	
2 y	0.43 (0.31-0.58)		0.65 (0.46-0.91)	
3 y	1.26 (0.96-1.65)		1.48 (1.11-1.96)	
Residential location				
Rural	1 [Reference]	.28	1 [Reference]	.44
Urban	1.12 (0.91-1.36)		1.09 (0.88-1.35)	
Low family income^c^	1.36 (1.11-1.67)	.004	1.32 (1.06-1.64)	.01
≥2 Children aged <5 y in household	2.28 (1.86-2.80)	.01	1.99 (1.60-2.48)	<.001
**PCV10 Serotype Pneumococcal Carriage (n = 1954)**^**d**^				
Infant mode of delivery				
Cesarean	1 [Reference]	.03	1 [Reference]	.17
Vaginal	2.21 (1.07-4.58)		1.67 (0.80-3.51)	
Survey year				
Before introduction of PCV10	1 [Reference]	.002	1 [Reference]	.03
After introduction of PCV10				
1 y	0.60 (0.37-0.96)		0.65 (0.39-1.07)	
2 y	0.34 (0.19-0.60)		0.41 (0.22-0.75)	
3 y	0.59 (0.36-0.96)		0.63 (0.38-1.04)	
Residential location				
Rural	1 [Reference]	.006	1 [Reference]	.02
Urban	1.70 (1.16-2.47)		1.64 (1.10-2.44)	
Low family income^c^	1.06 (0.73-1.54)	.77	0.99 (0.67-1.48)	.98
≥2 Children aged <5 y in household	1.94 (1.33-2.81)	.001	1.56 (1.04-2.32)	.03
**Non-PCV10 Serotype Pneumococcal Carriage (n = 1954)**^**e**^				
Infant mode of delivery				
Cesarean	1 [Reference]	.03	1 [Reference]	.04
Vaginal	1.52 (1.05-2.18)		1.49 (1.01-2.20)	
Survey year				
Before introduction of PCV10	1 [Reference]	<.001	1 [Reference]	<.001
After introduction of PCV10				
1 y	0.81 (0.59-1.12)		1.14 (0.81-1.61)	
2 y	0.52 (0.37-0.74)		0.85 (0.58-1.25)	
3 y	1.65 (1.23-2.22)		2.00 (1.45-2.73)	
Residential location				
Rural	1 [Reference]	.71	1 [Reference]	.49
Urban	0.96 (0.77-1.20)		0.92 (0.72-1.16)	
Low family income^c^	1.37 (1.09-1.71)	.007	1.36 (1.07-1.73)	.01
≥2 Children aged <5 y in household	2.39 (1.91-3.00)	<.001	2.25 (1.76-2.88)	<.001

Abbreviations: OR, odds ratio; PCV10, 10-valent pneumococcal conjugate vaccine.

^a^ Variables adjusted for were survey year, residential location, low family income, and 2 or more children younger than 5 years living in the household.

^b^ Any pneumococci, including nonencapsulated pneumococci.

^c^ Weekly family income below the basic needs poverty line (<FJ$175 per week).^42^

^d^ Pneumococci included in PCV10 (serotypes 1, 4, 5, 6B, 7F, 9V, 14, 18C, 19F, and 23F).

^e^ Pneumococcal serotypes not included in PCV10, including nonencapsulated pneumococci.

### Association Between Infant Mode of Delivery and Pneumococcal Density

eTable 3 in the Supplement shows unadjusted and adjusted differences in median density of overall, PCV10, and non-PCV10 pneumococci. We found no association between infant mode of delivery and pneumococcal density (overall, PCV10, and non-PCV10).

## Discussion

This study describes pneumococcal carriage by infant mode of delivery. Vaginal delivery was positively associated with overall and non-PCV10 carriage. The prevalence of overall, PCV10, and non-PCV10 carriage was greater in infants delivered vaginally, compared with those delivered by cesarean birth. Possible reasons for these differences in pneumococcal carriage by infant mode of delivery include vertical transmission of pneumococci, reduced acquisition of pneumococci owing to routine intrapartum administration of antibiotics administered during cesarean delivery for postoperative wound prophylaxis, and/or the association that infant mode of delivery may have with the infant microbiome.

We found a positive association between vaginal delivery and pneumococcal carriage, raising the hypothesis of a potential vertical component to pneumococcal acquisition for very young infants. Contact with toddlers is a risk factor for pneumococcal carriage via horizontal transmission.^27,28,29^ However, we found the positive association between vaginal delivery and pneumococcal carriage remained after adjustment for the potential of horizontal transmission from young children living in the same household. This finding suggests that vertical transmission may be a route for acquisition of nasopharyngeal pneumococci in very young infants, which has been proposed previously.^6,7^ Maternal vaginal colonization and subsequent transmission during delivery to infants is documented for other bacteria.^44^ The pneumococcus is not considered a commensal of the human vaginal tract, as the prevalence of pneumococcal vaginal colonization is very low.^16,22^ However, transient cervicovaginal colonization can occur, and vertical transmission of pneumococci may occur via premature or prolonged rupture of membranes, chorioamnionitis, or perinatal maternal pneumococcal disease.^6,16,22,26^ Case reports from high-income countries of neonatal IPD, as well as maternal pneumococcal vaginal colonization, suggest vertical transmission of pneumococci during delivery.^6,7^ Despite high rates of early infant IPD in LMICs, we were unable to find studies from LMICs on maternal vaginal pneumococcal carriage rates. We did not collect maternal vaginal swabs or nasopharyngeal swabs from children living in participants’ households, so we were unable to determine the route of transmission.

The association found between vaginal delivery and infant carriage may also be explained by receipt of maternal intrapartum antibiotics for postoperative wound prophylaxis for cesarean delivery and neonatal sepsis. A double-blind, placebo-controlled randomized trial of azithromycin given to women in labor in Gambia found substantial reductions in prevalence of pneumococcal carriage in infants aged 28 days born to women who received azithromycin, compared with those born to women who received placebo (prevalence ratio, 0.67 [95% CI, 0.53-0.83]; *P* < .001).^45^ However, perinatal azithromycin use is uncommon. In Fiji, flucloxacillin and amoxycillin are the usual antibiotics of choice for surgical wound and neonatal sepsis prophylaxis. We did not record which women received antibiotics, and were unable to stratify analyses by receipt of antibiotics during delivery. Nevertheless, maternal antibiotics may have affected infants born to women undergoing cesarean delivery either transplacentally and/or through breast milk ingestion after delivery, which may have reduced pneumococcal acquisition by vertical and/or horizontal routes.^46,47,48^

The differences we found in carriage by mode of delivery may be owing to the differential effects of mode of delivery on the development of the infant microbiome. The infant microbiome develops different profiles after vaginal or cesarean delivery, owing to exposure to maternal vaginal or environmental flora and the disruptive effects of intrapartum prophylactic antibiotics,^48,49^ which may affect pneumococcal carriage in very young infants. A longitudinal study of Dutch infants found that, after cesarean delivery, there was a delay in the development of the infant microbiome, with lower relative abundance of pneumococci up to 3 months of age, compared with those born vaginally.^50^

Exposure to environmental flora and intrapartum antibiotics during cesarean delivery may have mediated both the association between vaginal delivery and pneumococcal carriage and the number of unique serotypes detected, through their association with maternal and infant microbiomes. We found more pneumococcal serotypes from infants born vaginally compared with those delivered by cesarean birth. More important, serotypes 4 and 18C were found exclusively in infants delivered vaginally, and represented 9.3% of PCV10 serotypes found in such infants. This finding is important, as these serotypes are not included in the PCV10 vaccine (Pneumosil) currently undergoing phase 3 clinical trials in India.^51^ A World Health Organization review of the age distribution of pneumococcal disease found that, despite some uncertainty about the rates of pneumococcal infection in very young infants, 32% to 79% of serotypes causing IPD in infants younger than 2 months would be covered by PCV10 or PCV13.^2^ We found a lower percentage covered by PCV10 or PCV13, as our study was conducted up to 3 years after the introduction of PCV10. Nevertheless, despite evidence of PCV indirect effects on very young unvaccinated infants,^30^ many such infants will remain at risk of IPD due to nonvaccine serotypes.^3,52^ The difference found in number of serotypes by mode of delivery may be owing to the higher number of participants delivered vaginally compared with cesarean delivery (1722 vs 260, after excluding nasopharyngeal swab samples owing to insufficient volume, sample loss, or labeling errors), which may lend itself to a greater number of serotypes being detected in swab samples from vaginally born infants.

We found a positive association between infant mode of delivery and overall and non-PCV10 carriage. The 95% CI observed for PCV10 carriage, which crossed the null value, may be owing to the small number of infants carrying PCV10 serotypes, likely reflecting the timing of our study in relation to the introduction of PCV10 in Fiji. A previous study reported that PCV10 carriage declined among unvaccinated infants age 5 to 8 weeks after the introduction of PCV10, suggesting indirect protection.^30^ As such, the 95% CI estimated for the association between infant mode of delivery and PCV10 carriage may be explained by reduced rates of PCV10 carriage owing to herd immunity, and the small number of cesarean delivered infants who were PCV10 carriers (n = 8).

We found that carriage of multiple serotypes was uncommon and similar by infant mode of delivery. Few studies have examined rates of multiple-serotype carriage in this age group or used molecular serotyping methods to do so, and to our knowledge, no other study has compared the prevalence of multiple-serotype carriage by infant mode of delivery.

Although data are limited, estimates suggest the pneumococcus remains a significant pathogen with regard to neonatal sepsis in LMICs.^53^ A systematic review of the global burden of neonatal IPD estimated the pooled incidence in less-developed countries to be 16 (95% CI, 3.9-65.6) per 100 000 live births and in the more developed countries to be 41 (95% CI, 29.1-58.1) per 100 000.^53^ These counterintuitive estimates are likely driven by differential case ascertainment by setting.^53^ A review of the cause of community-acquired neonatal sepsis in LMICs isolated *Streptococcus pneumoniae* from 4.6% (95% CI, 2.1%-7.1%) of infants younger than 7 days and 5.2% (95% CI, 4.2%-6.3%) of infants ages 8 to 59 days.^3^ A study on the cause of serious infection in young infants in low-income countries found 8% of bacteremia in infants younger than 7 days to be due to pneumococci and 10.2% of bacteremia aged 8 to 29 days to be due to pneumococci.^5^ The same study found 38.5% of meningitis cases in infants aged 7 to 29 days to be due to pneumococcus. A global review of pneumococcal disease found that 21% of all pneumococcal meningitis cases in children younger than 5 years occurred in infants younger than 2 months.^2^ In addition, several cases of neonatal IPD, including sepsis, with pneumococci isolated from maternal genital tracts have been reported.^6,7^ Our findings suggest that, in Fiji, pneumococcus may be an important cause of young infant infection and should be considered in cases of infection in such a population in Fiji and other LMICs.

### Limitations

There were limitations to our study in addition to those mentioned earlier. First, generalizability may be limited to unvaccinated Fijian infants living in Suva. Because of the cross-sectional design, this study is unable to assume causality between infant mode of delivery and pneumococcal carriage. However, delivery precedes nasopharyngeal sampling, such that temporality regarding infant mode of delivery and carriage may be assumed. Data supporting an association between infant mode of delivery and pneumococcal carriage may be confounded by unmeasured clinical factors, such as prematurity.^26^ The possibility of chance findings owing to multiple testing exists. However, this study was conducted in a tropical setting in the Asian Pacific, where infant pneumococcal carriage rates are relatively high, and sufficient participants were delivered via cesarean birth to achieve a sample size sufficient to undertake this analysis. In addition, nasopharyngeal swabs were collected and processed in accordance with World Health Organization guidelines.^36^ Pneumococci were detected and quantified using sensitive and quantitative molecular methods, and few data were missing.^38^

## Conclusions

This study found that infant mode of delivery is positively associated with pneumococcal carriage. Our observations may be owing to differences in early pneumococcal carriage through differential exposure to the vaginal microbiota during delivery and the association of intrapartum antibiotics during cesarean delivery with the infant microbiome. Our results generate the hypothesis that vertical transmission may occur. Our findings have important public health and clinical implications for young infants in LMICs. As pneumococcal carriage rates are far higher in young infants in LMICs than those in high-income countries, rates of pneumococcal sepsis in young infants are likely considerable.^2^ Among neonates in LMICs, the burden of pneumococcal carriage and IPD is largely unknown, but, where reported, are substantial.^2^ Invasive pneumococcal disease should be considered in cases of neonatal sepsis in LMICs,^3^ as herd protection in unvaccinated infants cannot be assumed as current PCV formulations may not provide adequate coverage for many IPD serotypes.

In post-PCV settings, and with increasing consideration of reduced-dose PCV schedules, monitoring pneumococcal transmission will be central to pneumococcal disease control, particularly in the most vulnerable age groups.^54^ To our knowledge, there are no data on the prevalence of pneumococcal vaginal colonization in LMICs. Further research is required to investigate the possibility of vertical transmission during delivery, maternal pneumococcal vaginal colonization, antibiotic use during labor, the effect of mode of delivery on the infant microbiome (as are studies on neonatal carriage in other LMICs), and enhanced neonatal IPD surveillance.

## Notes

Supplement. **eFigure.** Directed Acyclic Graph to Select Factors for Statistical Adjustment **eAppendix.** Dagity Code for eFigure, Directed Acyclic Graph to Identify a Minimal Set of Factors for Statistical Adjustment **eTable 1.** Comparison of Serotype-Specific Carriage in Fijian Infants Aged 5 to 8 Weeks, by Infant Mode of Delivery **eTable 2.** Correlation Between Exposures Included in Regression Models Assessed Using Pearson *r* **eTable 3.** Associations Between Infant Mode of Delivery and Pneumococcal Carriage Density in Fijian Infants Aged 5 to 8 Weeks, in 4 Annual Pneumococcal Carriage Surveys **eReferences**

## References

[1] Wahl B, O’Brien KL, Greenbaum A, Burden of *Streptococcus pneumoniae* and *Haemophilus influenzae* type b disease in children in the era of conjugate vaccines: global, regional, and national estimates for 2000-15. Lancet Glob Health. 2018;6(7):-. doi:10.1016/S2214-109X(18)30247-X 29903376 PMC6005122

[2] Russell FM, Sanderson C, Temple B, Mulholland K Global Review of the Distribution of Pneumococcal Disease by Age and Region. Geneva, Switzerland: World Health Organization; 2011.

[3] Waters D, Jawad I, Ahmad A, Aetiology of community-acquired neonatal sepsis in low and middle income countries. J Glob Health. 2011;1(2):154-170. 23198116 PMC3484773

[4] Okike IO, Johnson AP, Henderson KL, ; neoMen Study Group Incidence, etiology, and outcome of bacterial meningitis in infants aged <90 days in the United kingdom and Republic of Ireland: prospective, enhanced, national population-based surveillance. Clin Infect Dis. 2014;59(10):e150-e157. doi:10.1093/cid/ciu514 24997051

[5] The WHO Young Infants Study Group Bacterial etiology of serious infections in young infants in developing countries: results of a multicenter study. Pediatr Infect Dis J. 1999;18(10)(suppl):S17-S22. 10530569 10.1097/00006454-199910001-00004

[6] Alsubaie SS Early-onset neonatal pneumococcal infection: a problem deserving more recognition: a case report and review of the literature. Infect Dis Clin Pract. 2019;27(2):68-72. doi:10.1097/IPC.0000000000000696

[7] Fothy JF, Vetter S, Iñigo A, Gil J, Pérez JL, Hervás JA Early-onset *Streptococcus pneumoniae* neonatal sepsis and meningitis in the 13-valent vaccine era. Pediatr Infect Dis J. 2013;32(11):1299-1300. doi:10.1097/INF.0b013e31829ebeea 24141804

[8] Weintraub MI, Otto RN Pneumococcal meningitis and endophthalmitis in a newborn. JAMA. 1972;219(13):1763-1764. doi:10.1001/jama.219.13.1763 4553451

[9] Rhodes PG, Burry VF, Hall RT, Cox R Pneumococcal septicemia and meningitis in the neonate. J Pediatr. 1975;86(4):593-595. doi:10.1016/S0022-3476(75)80159-4 236367

[10] Bortolussi R, Thompson TR, Ferrieri P Early-onset pneumococcal sepsis in newborn infants. Pediatrics. 1977;60(3):352-355. 19725

[11] Tarpay MM, Turbeville DF, Krous HF Fatal streptococcus pneumoniae type III sepsis in mother and infant. Am J Obstet Gynecol. 1980;136(2):257. doi:10.1016/0002-9378(80)90608-0 7352510

[12] Naylor JC, Wagner KR Neonatal sepsis due to *Streptococcus pneumoniae*. CMAJ. 1985;133(10):1019-1020. 4063901 PMC1346416

[13] Andreu A, Genover E, Coira A, Farrán I Antepartum infection as a result of *Streptococcus pneumoniae* and sepsis in neonate. Am J Obstet Gynecol. 1989;161(5):1424-1425. doi:10.1016/0002-9378(89)90724-2 2589470

[14] Geelen SP, Gerards LJ, Fleer A Pneumococcal septicemia in the newborn: a report on seven cases and a review of the literature. J Perinat Med. 1990;18(2):125-129. doi:10.1515/jpme.1990.18.2.125 2366133

[15] Wright ED, Lortan JE, Perinpanayagam RM Early-onset neonatal pneumococcal sepsis in siblings. J Infect. 1990;20(1):59-63. doi:10.1016/S0163-4453(90)92368-U 2299184

[16] Primhak RA, Tanner MS, Spencer RC Pneumococcal infection in the newborn. Arch Dis Child. 1993;69(3 Spec No):317-318. doi:10.1136/adc.69.3_Spec_No.317 8215574 PMC1029501

[17] Simpson JM, Patel JS, Ispahani P *Streptococcus pneumoniae* invasive disease in the neonatal period: an increasing problem? Eur J Pediatr. 1995;154(7):563-566. doi:10.1007/BF02074835 7556324

[18] McDonald LC, Bryant K, Snyder J Peripartum transmission of penicillin-resistant *Streptococcus pneumoniae*. J Clin Microbiol. 2003;41(5):2258-2260. doi:10.1128/JCM.41.5.2258-2260.2003 12734296 PMC154670

[19] Sakran W, Valinsky L, Koren A, Bor N, Yishai R, Colodner R Early onset of neonatal *Streptococcus pneumoniae* bacteremia and septic arthritis. Clin Pediatr (Phila). 2004;43(6):579-581. doi:10.1177/000992280404300613 15248014

[20] Sallam A, Paes B *Streptococcus pneumoniae*: an old bug with significant maternal–newborn implications. Am J Perinatol. 2004;21(8):491-495. doi:10.1055/s-2004-835967 15580546

[21] Karabayir N, Hatipoglu N, Adal E, Sanli K A rare case of sepsis in newborn: *Streptococcus pneumoniae* septicemia. Arch Gynecol Obstet. 2010;282(5):591-592. doi:10.1007/s00404-010-1489-y 20428879

[22] Darbás H, Boyer G Isolation of *Streptococcus pneumoniae* from genital samples: discussion of its pathogenic role [in French]. Pathol Biol (Paris). 1987;35(2):177-180. 3550628

[23] Simell B, Auranen K, Käyhty H, Goldblatt D, Dagan R, O’Brien KL; Pneumococcal Carriage Group The fundamental link between pneumococcal carriage and disease. Expert Rev Vaccines. 2012;11(7):841-855. doi:10.1586/erv.12.53 22913260

[24] Vu HT, Yoshida LM, Suzuki M, Association between nasopharyngeal load of *Streptococcus pneumoniae*, viral coinfection, and radiologically confirmed pneumonia in Vietnamese children. Pediatr Infect Dis J. 2011;30(1):11-18. doi:10.1097/INF.0b013e3181f111a2 20686433

[25] Aho C, Michael A, Yoannes M, ; Neonatal Pneumococcal Conjugate Vaccine Trial Study Team Limited impact of neonatal or early infant schedules of 7-valent pneumococcal conjugate vaccination on nasopharyngeal carriage of *Streptococcus pneumoniae* in Papua New Guinean children: a randomized controlled trial. Vaccine Rep. 2016;6:36-43. doi:10.1016/j.vacrep.2016.08.002 28580433 PMC5446595

[26] Hoffman JA, Mason EO, Schutze GE, *Streptococcus pneumoniae* infections in the neonate. Pediatrics. 2003;112(5):1095-1102. doi:10.1542/peds.112.5.1095 14595052

[27] Althouse BM, Hammitt LL, Grant L, Identifying transmission routes of *Streptococcus pneumoniae* and sources of acquisitions in high transmission communities. Epidemiol Infect. 2017;145(13):2750-2758. doi:10.1017/S095026881700125X 28847317 PMC5647670

[28] Weinberger DM, Pitzer VE, Regev-Yochay G, Givon-Lavi N, Dagan R Association between the decline in pneumococcal disease in unimmunized adults and vaccine-derived protection against colonization in toddlers and preschool-aged children. Am J Epidemiol. 2019;188(1):160-168. doi:10.1093/aje/kwy219 30462150 PMC6321804

[29] Neal EFG, Flasche S, Ratu T, Ethnicity and mixing with older children are risk factors for vaccine-type pneumococcal carriage post 10-valent pneumococcal conjugate vaccine introduction in Fiji: a cross-sectional study. Paper presented at: 11th International Symposium on Pneumococci and Pneumococcal Diseases; April 18, 2018; Melbourne, Australia.

[30] Dunne EM, Satzke C, Ratu FT, Effect of ten-valent pneumococcal conjugate vaccine introduction on pneumococcal carriage in Fiji: results from four annual cross-sectional carriage surveys. Lancet Glob Health. 2018;6(12):e1375-e1385. doi:10.1016/S2214-109X(18)30383-8 30420033 PMC6231327

[31] Government of Fiji, Fiji Islands Bureau of Statistics 2017 Population and Housing Census. Suva, Fiji: Government of Fiji; 2018.

[32] Ministry of Health Annual report 2013 https://www.health.gov.fj/wp-content/uploads/2018/03/Annual-Report-2013.pdf. Accessed November 11, 2018.

[33] Ministry of Health & Medical Services Ministry of Health & Medical Services annual report 2014 https://www.health.gov.fj/PDFs/Annual Report/Annual Report 2014.pdf. Accessed November 11, 2018.

[34] Ministry of Health & Medical Services Annual report 2015 http://www.parliament.gov.fj/wp-content/uploads/2017/02/MoHMS-AR-2015.pdf. Accessed November 11, 2018.

[35] Russell FM, Carapetis JR, Ketaiwai S, Pneumococcal nasopharyngeal carriage and patterns of penicillin resistance in young children in Fiji. Ann Trop Paediatr. 2006;26(3):187-197. doi:10.1179/146532806X120273 16925955

[36] Satzke C, Turner P, Virolainen-Julkunen A, ; WHO Pneumococcal Carriage Working Group Standard method for detecting upper respiratory carriage of *Streptococcus pneumoniae*: updated recommendations from the World Health Organization Pneumococcal Carriage Working Group. Vaccine. 2013;32(1):165-179. doi:10.1016/j.vaccine.2013.08.062 24331112

[37] Carvalho MdaG, Tondella ML, McCaustland K, Evaluation and improvement of real-time PCR assays targeting *lytA*, *ply*, and *psaA* genes for detection of pneumococcal DNA. J Clin Microbiol. 2007;45(8):2460-2466. doi:10.1128/JCM.02498-06 17537936 PMC1951257

[38] Satzke C, Dunne EM, Porter BD, Klugman KP, Mulholland EK; PneuCarriage project group The PneuCarriage Project: a multi-centre comparative study to identify the best serotyping methods for examining pneumococcal carriage in vaccine evaluation studies. PLoS Med. 2015;12(11):e1001903. doi:10.1371/journal.pmed.1001903 26575033 PMC4648509

[39] van Selm S, van Cann LM, Kolkman MAB, van der Zeijst BAM, van Putten JPM Genetic basis for the structural difference between *Streptococcus pneumoniae* serotype 15B and 15C capsular polysaccharides. Infect Immun. 2003;71(11):6192-6198. doi:10.1128/IAI.71.11.6192-6198.2003 14573636 PMC219561

[40] Manna S, Ortika BD, Dunne EM, A novel genetic variant of *Streptococcus pneumoniae* serotype 11A discovered in Fiji. Clin Microbiol Infect. 2018;24(4):428.e1-428.e7. doi:10.1016/j.cmi.2017.06.031 28736074 PMC5869949

[41] Ram N, Nusair PW, Fong J, Mohammadnezhad M, Andajari S Prevalence and characteristics of caesarean section (CS) among pregnant women who delivered at the Colonial War Memorial Hospital (CWMH) Suva, Fiji, 2016. Pacific J Rep Health. 2018;1(7):384-389. doi:10.18313/pjrh.2018.906

[42] Narsey W, Raikoti T, Waqavonovono E Preliminary report: poverty and household incomes in Fiji in 2008-09 (based on the 2008-09 Household Income and Expenditure Survey). http://www.spc.int/nmdi/Reports/Fiji_HIES_2008-09.pdf. Accessed April 26, 2019.

[43] Salter SJ, Hinds J, Gould KA, Variation at the capsule locus, cps, of mistyped and non-typable *Streptococcus pneumoniae* isolates. Microbiology. 2012;158(pt 6):1560-1569. doi:10.1099/mic.0.056580-0 22403189 PMC3541774

[44] Seale AC, Bianchi-Jassir F, Russell NJ, Estimates of the burden of group B streptococcal disease worldwide for pregnant women, stillbirths, and children. Clin Infect Dis. 2017;65(suppl 2):S200-S219. doi:10.1093/cid/cix664 29117332 PMC5849940

[45] Roca A, Oluwalana C, Bojang A, Oral azithromycin given during labour decreases bacterial carriage in the mothers and their offspring: a double-blind randomized trial. Clin Microbiol Infect. 2016;22(6):565.e1-565.e9. doi:10.1016/j.cmi.2016.03.005 27026482 PMC4936760

[46] Pacifici GM Placental transfer of antibiotics administered to the mother: a review. Int J Clin Pharmacol Ther. 2006;44(2):57-63. doi:10.5414/CPP44057 16502764

[47] Mathew JL Effect of maternal antibiotics on breast feeding infants. Postgrad Med J. 2004;80(942):196-200. doi:10.1136/pgmj.2003.011973 15082839 PMC1742963

[48] Gallacher DJ, Kotecha S Respiratory microbiome of new-born infants. Front Pediatr. 2016;4:10. doi:10.3389/fped.2016.00010 26942168 PMC4762994

[49] Dominguez-Bello MG, Costello EK, Contreras M, Delivery mode shapes the acquisition and structure of the initial microbiota across multiple body habitats in newborns. Proc Natl Acad Sci U S A. 2010;107(26):11971-11975. doi:10.1073/pnas.1002601107 20566857 PMC2900693

[50] Bosch AATM, Levin E, van Houten MA, Development of upper respiratory tract microbiota in infancy is affected by mode of delivery. EBioMedicine. 2016;9(C):336-345. doi:10.1016/j.ebiom.2016.05.031 27333043 PMC4972531

[51] US National Library of Medicine, National Institutes of Health. Phase 3 study of 10-valent pneumococcal conjugate vaccine (PNEUMOSIL) administered in a 2+1 schedule to healthy infants. https://clinicaltrials.gov/ct2/show/record/NCT03896477. Accessed April 24, 2019.

[52] Mount V, Burton C, Jackson C, Heffernan H, Best E Neonatal invasive pneumococcal disease: New Zealand experience in the era of pneumococcal vaccination. Aust N Z J Obstet Gynaecol. 2017;57(3):280-285. doi:10.1111/ajo.12512 27530965

[53] Billings ME, Deloria-Knoll M, O’Brien KL Global burden of neonatal invasive pneumococcal disease: a systematic review and meta-analysis. Pediatr Infect Dis J. 2016;35(2):172-179. doi:10.1097/INF.0000000000000955 26517330

[54] Flasche S, Van Hoek AJ, Goldblatt D, The potential for reducing the number of pneumococcal conjugate vaccine doses while sustaining herd immunity in high-income countries. PLoS Med. 2015;12(6):e1001839. doi:10.1371/journal.pmed.1001839 26057994 PMC4461163

