# Supplementary material for: A Comparison of Pneumococcal Nasopharyngeal Carriage in Very Young Fijian Infants Born by Vaginal or Cesarean Delivery
Source: JAMA Netw Open. 2019 Oct 18;2(10):e1913650. doi: 10.1001/jamanetworkopen.2019.13650 (PMC6813584; doi:10.1001/jamanetworkopen.2019.13650)
Supplement: Supplement. — eFigure. Directed Acyclic Graph to Select Factors for Statistical Adjustment eAppendix. Dagity Code for eFigure, Directed Acyclic Graph to Identify a Minimal Set of Factors for Statistical Adjustment eTable 1. Comparison of Serotype-Specific Carriage in Fijian Infants Aged 5 to 8 Weeks, by Infant Mode of Delivery eTable 2. Correlation Between Exposures Included in Regression Models Assessed Using Pearson r eTable 3. Associations Between Infant Mode of Delivery and Pneumococcal Carriage Density in Fijian Infants Aged 5 to 8 Weeks, in 4 Annual Pneumococcal Carriage Surveys eReferences [file jamanetwopen-2-e1913650-s001.pdf]

Supplementary Online Content

Neal EFG, Nguyen C, Ratu FT, et al. A comparison of pneumococcal nasopharyngeal carriage in very young Fijian infants born by vaginal or cesarean delivery. *JAMA Netw Open.* 2019;2(10):e1913650. doi:10.1001/jamanetworkopen.2019.13650

eFigure. Directed Acyclic Graph to Select Factors for Statistical Adjustment
eAppendix. Dagity Code for eFigure, Directed Acyclic Graph to Identify a Minimal Set of Factors for Statistical Adjustment
eTable 1. Comparison of Serotype-Specific Carriage in Fijian Infants Aged 5 to 8 Weeks, by Infant Mode of Delivery
eTable 2. Correlation Between Exposures Included in Regression Models Assessed Using Pearson *r*
eTable 3. Associations Between Infant Mode of Delivery and Pneumococcal Carriage Density in Fijian Infants Aged 5 to 8 Weeks, in 4 Annual Pneumococcal Carriage Surveys
eReferences

This supplementary material has been provided by the authors to give readers additional information about their work.

© 2019 Neal EFG et al. *JAMA Network Open.*

eFigure. Directed Acyclic Graph to Select Factors for Statistical Adjustment

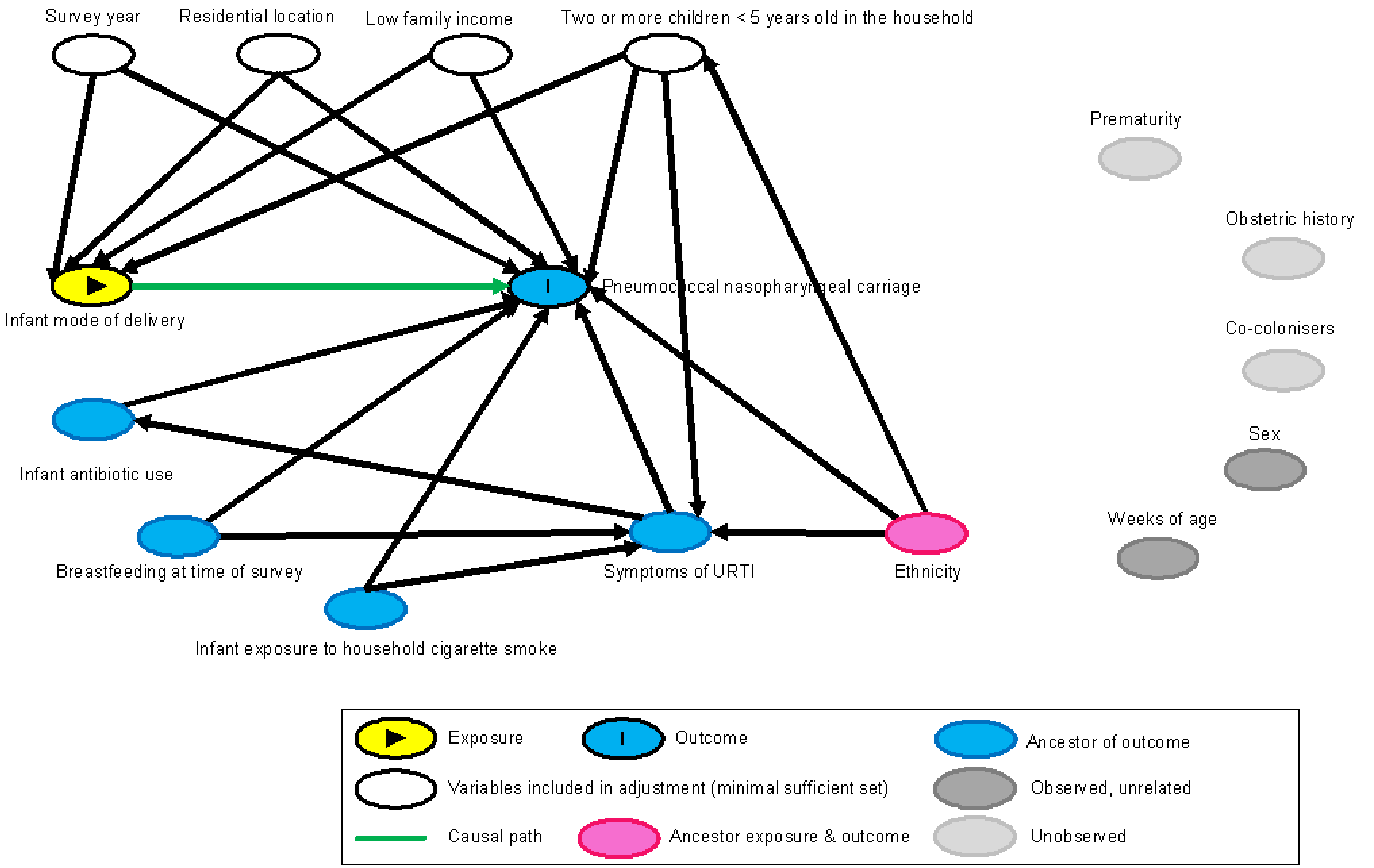

© 2019 Neal EFG et al. *JAMA Network Open.*

eAppendix. Dagity Code for eFigure, Directed Acyclic Graph to Identify a Minimal Set of Factors for Statistical Adjustment [1]

To recreate the directed acyclic graph in eFigure, please go to http://www.dagitty.net/dags.html# and copy and paste the code below in to the model code window on the bottom right of the screen, then select "Update Model", immediately below the model code. Finally, select "cancel" for the new variable option [1].

```
Breastfeeding%20at%20time%20of%20survey 1 @0.309,0.771
Co-colonisers U @0.579,0.451
Infant%20antibiotic%20use 1 @0.258,0.727
Infant%20ethnicity 1 @0.498,0.813
Infant%20exposure%20to%20household%20cigarette%20smoke 1 @0.376,0.815
Infant%20mode%20of%20delivery E @0.264,0.603
Low%20family%20income A @0.395,0.270
Obstetric%20history U @0.568,0.388
Pneumococcal%20nasopharyngeal%20carriage O @0.410,0.595
Prematurity U @0.550,0.330
Residential%20location A @0.324,0.262
Sex 1 @0.575,0.528
Survey%20year A @0.262,0.260
Symptoms%20of%20URTI 1 @0.433,0.742
Two%20or%20more%20children%20%3C%205%20years%20in%20the%20household A @0.482,0.270
Weeks%20of%20age 1 @0.552,0.593

Breastfeeding%20at%20time%20of%20survey Pneumococcal%20nasopharyngeal%20carriage
Symptoms%20of%20URTI
Infant%20antibiotic%20use Pneumococcal%20nasopharyngeal%20carriage
Infant%20ethnicity Pneumococcal%20nasopharyngeal%20carriage Symptoms%20of%20URTI
Two%20or%20more%20children%20%3C%205%20years%20in%20the%20household
Infant%20exposure%20to%20household%20cigarette%20smoke
Pneumococcal%20nasopharyngeal%20carriage Symptoms%20of%20URTI
Infant%20mode%20of%20delivery Pneumococcal%20nasopharyngeal%20carriage
Low%20family%20income Infant%20mode%20of%20delivery Pneumococcal%20nasopharyngeal%20carriage
Residential%20location Infant%20mode%20of%20delivery Pneumococcal%20nasopharyngeal%20carriage
Survey%20year Infant%20mode%20of%20delivery Pneumococcal%20nasopharyngeal%20carriage
Symptoms%20of%20URTI Infant%20antibiotic%20use Pneumococcal%20nasopharyngeal%20carriage
Two%20or%20more%20children%20%3C%205%20years%20in%20the%20household
Infant%20mode%20of%20delivery Pneumococcal%20nasopharyngeal%20carriage Symptoms%20of%20URTI
```

© 2019 Neal EFG et al. *JAMA Network Open.*

eTable 1. Comparison of Serotype-Specific Carriage in Fijian Infants Aged 5 to 8 Weeks, by Infant Mode of Delivery

Serotypes	Vaginal delivery<br>n = 1,722<br>n, % [95% CI]	Cesarean delivery<br>n = 260<br>n, % [95% CI]	*P*-value[a]
**PCV10 serotypes**			
1[b]	2, 0.12 [0.01–0.42]	-	-
4[b]	6, 0.35 [0.13–0.76]	-	-
6B[b]	26, 1.51 [0.99–2.20]	2, 0.77 [0.09–2.75]	.57
7F[b]	7, 0.41 [0.16–0.84]	-	-
9V[b]	6, 0.35 [0.13–0.76]	-	-
14[b]	9, 0.52 [0.24–0.99]	-	-
18C[b]	5, 0.29 [0.09–0.68]	-	-
19F[b]	34, 1.97 [1.37–2.75]	3, 1.15 [0.24–3.33]	.47
23F[b]	23, 1.34 [0.85–2.00]	3, 1.15 [0.24–3.33]	1.0
**Non-PCV10 serotypes**			
3	5, 0.29 [0.09–0.67]	-	
6A[b]	23, 1.34 [0.85–2.00]	3, 1.15 [0.24–3.33]	1.0
6C	18, 1.05 [0.62–1.65]	3, 1.15 [0.24–3.33]	.74
6D	9, 0.52 [0.24–0.99]	1 0.38 [0.01–2.12]	1.0
7B	2, 0.12 [0.01–0.42]	-	-
7C	9, 0.52 [0.24–0.99]	2, 0.77 [0.09–2.75]	.65
8	1, 0.06 [0.00–0.32]	-	-
10A	1, 0.06 [0.00–0.32]	-	-
10B	4, 0.23 [0.06–0.59]	1, 0.38 [0.01–2.12]	.51
11A	19, 1.10 [0.67–1.72]	2, 0.77 [0.09–2.75]	1.0
12F	2, 0.12 [0.01–0.42]	-	-
13	20, 1.16 [0.71–1.79]	2, 0.77 [0.09–2.75]	.75
15A	1, 0.06 [0.00–0.32]	-	-
15B/C	39, 2.26 [1.62–3.08]	6, 2.31 [0.85–4.95]	1.0
16F	31, 1.80 [1.23–2.55]	1, 0.38 [0.01–2.12]	.11
17F	13, 0.75 [0.40–1.29]	2, 0.77 [0.09–2.75]	1.0
18A	5, 0.29 [0.09–0.68]	-	-
19A[b]	19, 1.10 [0.67–1.72]	1, 0.38 [0.01–2.12]	.50
19B	6, 0.35 [0.13–0.76]	-	-
20	3, 0.17 [0.04–0.51]	-	-
20B	2, 0.12 [0.01–0.42]	-	-
21	16, 0.93 [0.53–1.50]	5, 1.95 [0.63–4.43]	.18
22F	8, 0.46 [0.20–0.91]	1, 0.38 [0.01–2.12]	1.0
23A	14, 0.81 [0.45–1.36]	-	-
23B	6, 0.35 [0.13–0.76]	-	-
24B	2, 0.12 [0.01–0.42]	1, 0.38 [0.01–2.12]	.34
24F	5, 0.29 [0.09–0.68]	-	-
29	2, 0.12 [0.01–0.42]	-	-
31	5, 0.29 [0.09–0.68]	-	-
33A	1, 0.06 [0.00–0.32]	-	-
33B	5, 0.29 [0.09–0.68]	1, 0.38 [0.01–2.12]	.57
33F	3, 0.17 [0.04–0.51]	-	-

© 2019 Neal EFG et al. *JAMA Network Open.*

Serotypes	Vaginal delivery (n = 1,722) n, (%) [95% CI]	Cesarean delivery (n = 260) n, (%) [95% CI]	*P*-value[a]
34	15, 0.87 [0.49–1.43]	2, 0.77 [0.09–2.75]	1.0
35A	7, 0.41 [0.16–0.84]	-	-
35B	23, 1.38 [0.85–2.00]	1, 0.38 [0.01–2.12]	.35
35F	1, 0.06 [0.00–0.32]	-	-
38	5, 0.29 [0.09–0.68]	1, 0.38 [0.01–2.12]	.57
39	4, 0.23 [0.06–0.59]	-	-
NT2	22, 1.28 [0.80–1.93]	2, 0.77 [0.09–2.75]	.76
NT2/NT3b	14, 0.81 [0.45–1.36]	2, 0.77 [0.09–2.75]	1.0
NT3b	16, 0.93 [0.53–1.50]	4, 1.54 [0.42–3.89]	.32
NT4	4, 0.23 [0.06–0.59]	-	-

Abbreviation: CI Confidence interval; [a] *P*-values were determined from Fisher's exact test; [b] Serotypes included in PCV13

© 2019 Neal EFG et al. *JAMA Network Open.*

eTable 2. Correlation Between Exposures Included in Regression Models Assessed Using Pearson *r*

Characteristics	Infant mode of delivery	Survey year	Residential location	Low family income	≥ 2 children < 5 y in the household
**Infant mode of delivery**	N / A	-0.0887	0.1205	-0.0553	0.0794
**Survey year**	-0.0887	N / A	-0.0533	0.0913	-0.0342
**Residential location**	0.1205	-0.0533	N / A	-0.0867	0.1569
**Low family income**	-0.0553	0.0913	-0.0867	N / A	-0.0083
**≥ 2 children < 5 y in the household**	0.0794	-0.0342	0.1569	-0.0083	N / A

© 2019 Neal EFG et al. *JAMA Network Open.*

**eTable 3.** Associations Between Infant Mode of Delivery and Pneumococcal Carriage Density in Fijian Infants Aged 5 to 8 Weeks, in 4 Annual Pneumococcal Carriage Surveys				
**Characteristics**	**Unadjusted Model**		**Adjusted Model[a]**	
	**Differences in medians (95% CI)**	***P* Value**	**Differences in medians (95% CI)**	***P* Value**
**Overall pneumococcal density ($\log_{10}$ GE/ml)[b] (n=516)**				
Infant mode of delivery				
Cesarean	1 [Reference]		1 [Reference]	
Vaginal	0.41 (-0.05, 0.87)	.08	0.27 (-0.16, 0.70)	.22
Survey year				
Before introduction of PCV10	1 [Reference]		1 [Reference]	
After introduction of PCV10				
1 y	-0.04 (-0.38, 0.29)	<.001	0.07 (-0.28, 0.43)	<.001
2 y	-0.20 (-0.58, 0.18)		-0.12 (-0.41, 0.37)	
3 y	-0.91 (-1.21, -0.60)		-0.68 (-1.00, -0.36)	
Residential location				
Rural	1 [Reference]		1 [Reference]	
Urban	0.34 (0.10, 0.58)	.006	0.13 (-0.11, 0.38)	.13
Low family income[c]	-0.05 (-0.33, 0.23)	.74	-0.04 (-0.28, 0.21)	.77
≥ 2 children < 5 y in the household	0.25 (-0.01, 0.52)	.07	0.24 (-0.01, 0.50)	.06
**PCV10 serotype pneumococcal density ($\log_{10}$ GE/ml)[d] (n=121)**				
Infant mode of delivery				
Cesarean	1 [Reference]		1 [Reference]	
Vaginal	0.44 (-0.64, 1.53)	.42	0.10 (-0.92, 1.11)	.85
Survey year				
Before introduction of PCV10	1 [Reference]		1 [Reference]	
After introduction of PCV10				
Pre-PCV10	1 [Reference]		1 [Reference]	
1 y	-0.28 (-0.98, 0.43)	.06	-0.24 (-0.91, 0.43)	.15
2 y	-0.70 (-1.55, 0.16)		-0.56 (-1.37, 0.25)	
3 y	-0.85 (-1.57, -1.27)		-0.75 (-1.43, 0.-07)	
Residential location				
Rural	1 [Reference]		1 [Reference]	
Urban	0.31 (-0.23, 0.85)	.26	0.21 (-0.32, 0.75)	.43
Low family income[c]	0.11 (-0.46, 0.67)	.71	-0.08 (-0.60, 0.44)	.77
≥ 2 children < 5 y in the household	-0.13 (-0.70, 0.45)	.66	0.15 (-0.38, 0.69)	.57
**Non-PCV10 serotype pneumococcal density ($\log_{10}$ GE/ml)[e] (n=393)**				
Infant mode of delivery				
Cesarean	1 [Reference]		1 [Reference]	
Vaginal	0.51 (-0.01, 1.03)	.06	0.12 (-0.34, 0.60)	.60
Survey year				
Before introduction of PCV10	1 [Reference]		1 [Reference]	
After introduction of PCV10				
1 y	0.09 (0.29, 0.50)	<.001	0.17 (-0.25, 0.58)	<.001
2 y	-0.03 (-0.45, 0.39)		0.08 (-0.38, 0.53)	
3 y	-0.86 (-1.20, -0.53)		-0.77 (-1.14, -0.39)	
Residential location				
Rural	1 [Reference]		1 [Reference]	
Urban	0.32 (0.05, 0.61)	.02	0.19 (-0.8, 0.46)	.17
Low family income[c]	-0.05 (-0.39, 0.28)	.76	-0.11 (-0.39, 0.17)	.45
≥ 2 children < 5 y in the household	0.19 (-0.14, 0.53)	.26	0.23 (-0.05, 0.52)	.11

© 2019 Neal EFG et al. *JAMA Network Open.*

Abbreviations: PCV10, ten-valent pneumococcal conjugate vaccine; CI, confidence interval
[a] Variables adjusted for were survey year, residential location, and low family income.
[b] Only includes participants who were pneumococcal carriers, including non-encapsulated pneumococci
[c] Weekly family income below the basic needs poverty line (<FJ$175 per week)[2].
[d] Only includes participants who were carriers of serotypes included in PCV10 (serotypes 1, 4, 5, 6B, 7F, 9V, 14, 18C, 19F, and 23F).
[e] Only includes participants who were carriers of serotypes not included in PCV10, including non-encapsulated pneumococci.

© 2019 Neal EFG et al. *JAMA Network Open.*

eReferences

1. Textor J, Hardt J, Knüppel S. DAGitty. *Epidemiology* 2011; **22**(5): 745.
2. Narsey W, Raikoti T, Waqavonovono E. Preliminary Report: Poverty and Household Incomes in Fiji in 2008-09 (based on the 2008-09 Household Income and Expenditure Survey): Fiji Islands Bureau of Statistics; 2010.

© 2019 Neal EFG et al. *JAMA Network Open.*
